# Integrated robotics platform with haptic control differentiates subjects with Parkinson’s disease from controls and quantifies the motor effects of levodopa

**DOI:** 10.1186/s12984-019-0598-5

**Published:** 2019-10-26

**Authors:** Pauline Gaprielian, Stephen H. Scott, Catherine Lowrey, Stuart Reid, Giovanna Pari, Ron Levy

**Affiliations:** 1 Centre for Neuroscience Studies, Queen’s University, Kingston, Ontario K7L 3N6 Canada; 2 Department of Biomedical and Molecular Sciences, Queen’s University, Kingston, Ontario K7L 3N6 Canada; 3 Department of Medicine, Queen’s University, Kingston General Hospital, Kingston, Ontario Canada; 4 Department of Surgery, Queen’s University, Kingston General Hospital, Kingston, Ontario Canada

**Keywords:** Robotic assessment, Parkinson’s disease, Dopamine replacement therapy

## Abstract

**Background:**

The use of integrated robotic technology to quantify the spectrum of motor symptoms of Parkinson’s Disease (PD) has the potential to facilitate objective assessment that is independent of clinical ratings. The purpose of this study is to use the KINARM exoskeleton robot to (1) differentiate subjects with PD from controls and (2) quantify the motor effects of dopamine replacement therapies (DRTs).

**Methods:**

Twenty-six subjects (Hoehn and Yahr mean 2.2; disease duration 0.5 to 15 years) were evaluated OFF (after > 12 h of their last dose) and ON their DRTs with the Unified Parkinson’s Disease Rating Scale (UPDRS) and the KINARM exoskeleton robot. Bilateral upper extremity bradykinesia, rigidity, and postural stability were quantified using a repetitive movement task to hit moving targets, a passive stretch task, and a torque unloading task, respectively. Performance was compared against healthy age-matched controls.

**Results:**

Mean hand speed was 41% slower and 25% fewer targets were hit in subjects with PD OFF medication than in controls. Receiver operating characteristic (ROC) area for hand speed was 0.94. The torque required to stop elbow movement during the passive stretch task was 34% lower in PD subjects versus controls and resulted in an ROC area of 0.91. The torque unloading task showed a maximum displacement that was 29% shorter than controls and had an ROC area of 0.71. Laterality indices for speed and end total torque were correlated to the most affected side. Hand speed laterality index had an ROC area of 0.80 against healthy controls. DRT administration resulted in a significant reduction in a cumulative score of parameter Z-scores (a measure of global performance compared to healthy controls) in subjects with clinically effective levodopa doses. The cumulative score was also correlated to UPDRS scores for the effect of DRT.

**Conclusions:**

Robotic assessment is able to objectively quantify parkinsonian symptoms of bradykinesia, rigidity and postural stability similar to the UPDRS. This integrated testing platform has the potential to aid clinicians in the management of PD and help assess the effects of novel therapies.

## Background

Parkinson’s Disease (PD) is a common neurodegenerative disorder that is characterized by bradykinesia/akinesia, rigidity, tremor and postural instability. Non-motor complications include autonomic and cognitive dysfunction [21]. In order to manage these symptoms, patients are treated with dopamine replacement therapies (DRTs) [10]. The progression of PD and the effects of therapy are assessed using the Unified Parkinson’s Disease Rating Scale (UPDRS). Part III of this scale results in a composite score of categorical variables for motor signs. However, the sensitivity of the UPDRS to the effect of therapies may be limited by inter-rater reliability [30], test-retest reliability [15], sensitivity [18], and bias [17].

Objective assessment technologies have the potential to complement the UPDRS and improve the clinical management of PD by providing greater uniformity between different physicians, care institutions and patients [25, 27, 37]. Devices that characterize the spectrum of signs in PD can be used to accurately distinguish patients with PD from healthy controls [1] and have a high test-retest reliability [5]. A major challenge of implementing assessment technologies for PD is to bridge the gap between complex behavioural data and clinical usefulness [13].

KINARM (BKIN Technologies, Kingston, Canada) is a robot-based assessment system that has been extensively used to measure neurological function in subjects with stroke [3, 38]. The system provides an integrated robotics and virtual reality platform and haptic control, permitting assessment of a broad range of sensory, motor, and cognitive functions. In this study we used integrated platform to objectively assess subjects with PD. Using tasks to specifically quantify bradykinesia, rigidity, and postural stability we determined whether this technology could (1) differentiate subjects with PD from healthy controls and (2) quantify the effects of anti-parkinsonian medication compared to UPDRS scores. Importantly, subject performance in each task can be compared to healthy controls. Discriminating abnormal from normal behavior is how clinicians diagnose and manage neurologic diseases. All items in the UPDRS differentiate healthy performance (rated as 0) from mild to severe/marked. Implicit in this scale is the concept that the rater makes a value judgement on how categorically far away a subject’s performance is from healthy performance. They compare a patient’s rigidity/bradykinesia/etc. to the distribution of healthy performance measures based on their past experience. These categorical scores become independent of units and therefore can be summed to achieve a total score. Similarly, we quantified subject performance in Z-space in order to calculate a cumulative score of the disparate tests performed with the robotic system.

## Methods

### Participant groups

Participants with PD were recruited from a movement disorders clinic at Kingston Health Sciences Centre in Kingston, Ontario, Canada. Inclusion criteria for this study were a diagnosis of idiopathic PD undergoing treatment with DRTs, the ability to understand the clinical and robotic assessment tasks, normal or corrected to normal vision, and no injury limiting movement of the upper extremity. Robotic task performance for each subject was compared against age- and handedness-matched healthy control subjects (using group means with significance set at α = 0.01). Two hundred fifty controls were used for the *object hit* analysis (mean age 62 years, range 43–75), 174 controls for the *object hit and avoid* analysis (mean age 64 years, range 47–75), 27 controls for the *elbow stretch task* analysis (mean age 59 years, range 35–79), and 38 controls for the *unloading task* analysis (mean age 63 years, range 49–80) (see below for task descriptions). The discrepancy in the number of control subjects is due to control data being collected before and during the recruitment of study subjects and that the control participants did not necessarily perform all of the tasks. Controls subjects were carefully screened by the research assistant, data analyst, and BKIN technologies to exclude diagnoses with a disease or condition affecting the brain, spinal cord, or vision, and musculoskeletal diseases and injuries. In addition, healthy subjects with abnormal behaviour on one or more standard tasks as noted by Z-scores > 2 on multiple parameters on more than one standard task were excluded. In total ~ 5% of recruits were excluded from the control data. This study was approved by Queen’s University and the Kingston General Hospital Research Institute ethics board and informed consent was obtained from each subject.

### Clinical assessment of Parkinson’s disease

Subjects with PD were instructed to stop taking their DRT medications after 6 pm the day before testing. One of two movement disorders neurologists administered Part III of the UPDRS during this “OFF” medication condition (SR tested 14 subjects, GP tested 12 subjects). The neurologists were not blinded to medication state. Subjects then completed the robotic test battery which took 15–20 min (see below). Upon completion, subjects were instructed to take their regularly scheduled dosages of DRTs. An hour rest period was given to each subject in order for their medications to be effective. The subjects then repeated the test battery and a movement disorders neurologist conducted Part III of the UPDRS to yield “ON” condition scores. Subjects were screened for dementia using the Montreal Cognitive Assessment (MoCA) scale during their ON condition. MoCA scores ranged from 20 to 30. Handedness was determined using the Edinburgh Handedness Inventory. The UPDRS and MoCA were not performed on the healthy controls.

### Robotic assessment

**Fig. 1:**
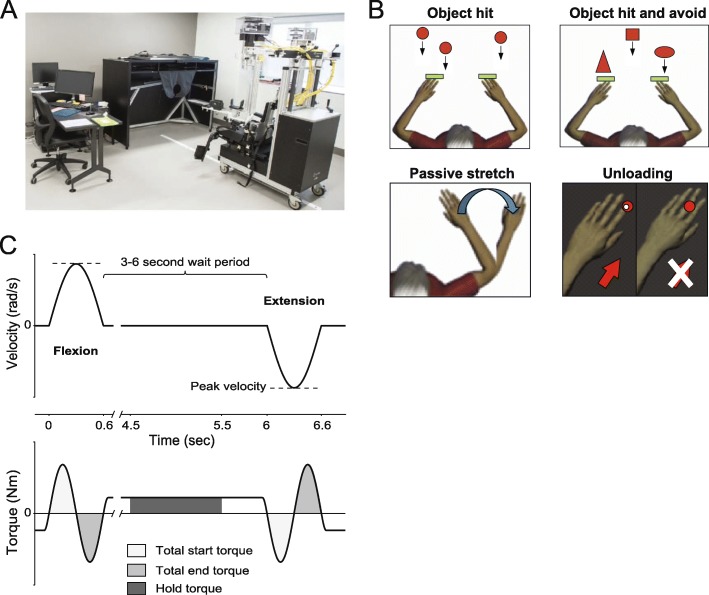
KINARM exoskeleton robot, tasks, and methodology to quantify rigidity. **a** The KINARM exoskeleton robot and virtual workspace used to quantify subject performance. Data collection is collected on the computer to the left of the KINARM virtual workspace. **b** Illustrations of the task battery for PD assessment. In the *object hit task* (top row, left panel) the subjects had to hit the red circles. In the *object hit and avoid task* (top row, right panel) subjects had to avoid hitting certain shapes (e.g. hit only squares and vertical ovals). The *passive stretch task* (bottom row, left panel) quantified rigidity by passively moving the arm at the elbow and recording the required torques (e.g. blue arrow for extension). In the *unloading task* (bottom row, right panel) the subject initially resists a joint torque (red arrow) and then the joint torque is suddenly removed (white X) and the subject has to maintain their arm posture by moving their hand back to the central target as quickly as possible. **c** A schematic of how the *passive stretch task* was used to model PD rigidity by moving elbow through a range of velocities (upper panel)

Robotic assessments were performed with the bilateral KINARM exoskeleton (Fig. 1a; BKIN Technologies, Kingston, Canada; Scott, 1999 [36]). This device permits arm movement in the horizontal plane, involving elbow and shoulder motion, and provides haptic feedback or moves the limb in the workspace. A virtual reality system projects visual feedback onto the horizontal workspace. A black screen is used to block the subject’s sight of their arms. Hand position is represented by a white dot or horizontal paddle dependent on the task. The robotic testing battery consists of four sequential tasks (Fig. 1b)*.* The *object hit task* and the *object hit and avoid task* were repetitive movement tasks that quantified bradykinesia and inhibitory control. Rigidity was quantified using the *passive stretch task*. Postural stability was quantified using a torque *unloading task*. The tasks are described in detail below.

Bradykinesia was quantified with two repetitive movement tasks that differed in complexity by the introduction of a cognitive load. The first was a simple repetitive reaching task called the *object hit task* and the second task, called the *object hit and avoid task*, required subjects to perform the same movements but to avoid hitting specific object shapes. In the *object hit task*, targets (2 cm diameter red balls) fell from the top of the viewing area towards the subjects (Fig. 1b, top row left panel). Subjects were instructed to hit away as many targets as possible within the task duration using 5 cm virtual paddles that corresponded to the position of their right and left hands [41]. Subjects could hit these balls with either hand. At the start of the task, a single falling ball moving at 10 cm/s was visible in the workspace. By the end of the task a maximum of 16 balls moving at 50 cm/sec were present on the screen. A total of 300 balls were released into the workspace falling from 10 different locations across the workspace. Haptic feedback was provided for correct target hits. The total duration of this task was ~ 2.5 min.

The *object hit and avoid task* was used to complement the *object hit task* in the assessment of bradykinesia in order to determine if a higher cognitive load worsened motor performance. It has been previously reported that bradykinesia is negatively associated with cognitive flexibility, in subjects with PD [35]. The objective of the *object hit and avoid task* was to hit as many ‘targets’ as possible while avoiding ‘distractors’. Two specific shapes were outlined as task targets at the start of the task. All other shapes were classified as distractors [4] (Fig. 1b, top row right panel). At the start of the task targets moved at 10 cm/s and increased to 50 cm/s by the end. A total of 200 targets and 100 distractors were released in the workspace from 10 different spatial locations. Haptic feedback was provided for correct target hits but not provided for distractor hits (i.e. trial errors). The total duration of this task was ~ 2.5 min.

The *passive stretch task* quantified rigidity over a continuous range at 2 different velocities (Fig. 1b, bottom row, left panel). The robotic system passively moved the elbow through flexion and extension between the angles of 65^o^ and 145^o^ while the shoulder was fixed at 60^o^ [8]. A single trial involved a flexion movement from 145^o^ to 65^o^, a 3 to 6 s wait period and an extension movement from 65^o^ back to 145^o^ [8]. At the beginning and end of each movement, torque could continue to be applied (blue arrow in Fig. 1b) in order to push the joint towards the desired location (i.e. some subjects exhibited baseline tone during the wait period). Each flexion and extension movement followed a bell-shaped velocity profile using a torque to accelerate the limb in a given direction and torque in the opposite direction of limb motion to slow down the limb at the end joint angle (see Fig. 1c). There were 5 flexion movements and 5 extension movements at two different movement times (600 ms and 1500 ms). Each elbow was tested separately with a total assessment time of ~ 2 min.

Lastly, the *unloading task* was used to model postural instability by testing how subjects corrected for a sudden change in upper limb posture (Fig. 1b, bottom row, right panel). The task began with a subject holding the white dot representative of their hand on a central red target [24]. A constant torque was applied to the elbow (±0.5 Nm) or shoulder (±1 Nm). The subject was asked to resist the load in order to maintain the cursor inside the central target. After a brief and random delay, the constant torque was quickly removed causing a displacement in the tested limb. Participants were instructed to move the hand back to the central target as quickly as they could. Visual feedback of the hand cursor was provided for the first 8 trials and afterwards, the hand cursor was extinguished at the onset of the unloading, requiring participants to use proprioception to return the hand back to the target location. There were 16 trials for each load condition presented in a random block design. Each arm was tested separately and total assessment time was ~ 8 min.

### Data analysis

Robotic data was recorded online with the KINARM embedded software (Dexterit-E version 3.5.4, BKIN Technologies, Kingston, Canada). The software automatically quantified subject performance using a number of parameters capturing spatial or temporal features of each task. These parameters were converted into normalized Z-scores based on performance of a large cohort of healthy controls and included the influence of sex, age and handedness [40]. Statistical analysis was performed using Sigmaplot (Version 11.0, Systat Software Inc.) and Matlab (version R2015b, MathWorks). In subjects with PD, a total UPDRS part III score, a bradykinesia score (sum of items 23–26; maximum of 32 points), an upper extremity bradykinesia score (sum of items 23, 24, 25; maximum of 12 points for each side), a rigidity score (sum of item 22; maximum of 20 points), an upper extremity rigidity score (item 22, maximum of 4 points for each side), and a posture score (item 30) were calculated for the OFF and ON conditions. The mean hand performance for the right and left hands were averaged for each parameter. All analyses except the asymmetry analysis incorporated the averaged parameter values. In order to determine body side asymmetries for bradykinesia and rigidity, the right minus left side difference between UPDRS items 22–26 were calculated when subjects were OFF medication [34].

In all subjects who performed *object hit* and *object hit and avoid tasks* to quantify bradykinesia, mean hand speed (m/s), total target hits, and movement area (area covered by both hands in the workspace, m^2^) were calculated [4, 41]. The total distractors hit were also considered in the *object hit and avoid task*.

In PD, increased rigidity requires more torque to move the limb and less torque to decelerate to a stop. Parkinsonian velocity-independent rigidity was calculated from the *passive stretch task* by integrating the torque (Nm) required to accelerate the forearm to the maximum velocity (“start total torque”) and then decelerate it back to rest (“end total torque”) (Fig. 1c). “Hold torque” was defined as the integrated torque required to maintain no movement for 1 s during the wait period (from 1.5–0.5 s before start of movement). This was measured to determine whether subjects were resisting the robot at the end points of movement and displaying passive rigidity [39]. Peak speed (rad/s) of the forearm was also calculated to assess for velocity dependent increases in tone. The start/end total torques, hold torque, and peak speeds were calculated for each flexion and extension movement for each arm (Fig. 1c). Preliminary analysis revealed that the parameters were most effective at identifying rigidity for the 600 ms (i.e faster) stretches (see Additional file 1), therefore, the results are presented for that movement time only. Values for flexion and extension movements were averaged.

To quantify postural stability in the *unloading task* the deceleration time (ms), maximum displacement (m), return time (ms) and endpoint error (m) of the cursor representing the hand position were calculated [24]. The maximum displacement was considered as the distance the hand traveled away from the red target upon the load release. Deceleration time represented how quickly the subject reacted to the load release. Return time was defined as how quickly the subject was able to return to the red target after the maximum displacement was reached. Finally, endpoint error was calculated by determining the hand distance from the centre red target at the end of the movement. Mean values for the right and left hands were averaged for each parameter.

### Statistical tests

Comparisons between groups were made with Student’s t-test and in case of non-normal data Mann-Whitney Rank Sum Test. The paired t-test or the Wilcoxon Signed Rank Test was used with paired data. Normality was determined using the Shapiro-Wilk test. Receiver operating characteristic (ROC) curves were used to assess the predictive accuracy of the KINARM test battery parameters to discriminate subjects with PD from healthy controls. A high discriminatory ability is represented by an area under the curve (AUC) that approaches 1 while a test that cannot differentiate the test populations has an AUC near 0.5. Differences between ROC curves were compared with the Chi-square test.

To quantify side-to-side asymmetry in PD during bimanual tasks, asymmetrical robotic performance between arms was quantified using a laterality index that was defined as the absolute difference between right arm and left arm parameters divided by the sum of both arms (Scharoun et al., 2015). ROC analysis for asymmetry was performed with absolute values of laterality indices and most affected UPDRS side.

Correlations of robotic measures with UPDRS scores, DRT dose, and MOCA scores were performed using Spearman Rank Order Correlation. Significance was set at *p* <  0.05.

A cumulative score was used as a measure of global performance in subjects with PD versus healthy controls and to facilitate comparisons with summed UPDRS bradykinesia, rigidity, and posture categorical item scores. Z-scores were first calculated for each task parameter based on performance from a large cohort of healthy control subjects [40]. The cumulative score was determined by calculating the root-sum-square distance of Z-scores for parameters within each task that showed a significant effect of DRT. We used the robotic parameters that showed a significant effect of DRT in the analysis since they are more sensitive to changes in PD state and can differentiate patients from control and OFF and ON states.

## Results

Twenty-six participants diagnosed with idiopathic PD undergoing treatment with DRTs were recruited from a movement disorders clinic. Subject characteristics are shown in Table 1. Subjects were between ages 47 and 75 years old (mean: 64.9, standard deviation: 8.7, SD) with their disease duration ranging from 0.5 to 15 years (mean: 5.5 years, SD: 3.4). The Hoehn and Yahr stage range was 2–3 (mean: 2.2, SD: 3.4). There were 22 right hand and 4 left hand dominant subjects. Mean levodopa dosage was 750 mg (range: 300 – 1375 mg). In addition to levodopa, seven subjects were taking pramipexole, three entacapone, one amantadine, one rasagiline, one trihexyphenidyl, one propranolol, and one with a rotigotine patch.

**Table 1:** Subject Characteristics

Subject	Age (yrs)	Sex	Disease duration (yrs)	Levodopa dose (mg)	Other parkinsonian medications	Other drug dosages (mg)	UPDRS motor OFF	UPDRS motor ON	MoCA
PD01	48	M	0.5	600	None	0	21	12	26
PD02	55	M	6	600	None	0	26	12	25
PD03^a^	71	M	5	800	Entacapone	400	54	26	21
PD04	67	M	5	700	Rasagiline, Pramipexole	1, 0.25	22	11	27
**PD05** ^a^	70	M	15	700	Entacapone	400	34	38	24
PD06^a^	67	M	11	400	Amantadine	200	46	13	22
**PD07**	55	M	3	550	None	0	30	24	30
PD08	72	M	13	350	None	0	33	22	27
**PD09**	57	M	4	600	None	0	12	7	27
PD10^a^	69	M	6	875	None	0	29	16	26
**PD11**	72	M	8	450	Pramipexole	1	28	22	26
PD12	70	F	5	650	Entacapone	200	28	10	28
PD13	53	F	2	1100	None	0	48	29	25
PD14	61	F	3	300	None	0	24	15	29
PD15	73	M	3	600	Pramipexole	3	18	7	25
**PD16** ^a^	75	M	8	500	Pramipexole	4.5	23	21	26
PD17	69	F	5	600	Pramipexole	1	38	27	25
PD18	72	M	5	1000	Pramipexole	3	33	17	20
PD19^a^	68	M	5	1050	Pramipexole	0.75	42	16	25
**PD20**	75	M	3	950	None	0	8	5	27
PD21^a^	58	M	5	600	Trihexyphenidyl	3	20	5	24
PD22^a^	54	F	4	450	Propranolol	60	27	16	26
PD23^a^	75	F	8	600	None	0	37	22	25
PD24	47	M	3	450	None	0	27	11	26
PD25^a^	64	M	6	1000	Rotigotine patch	4	35	6	21
PD26	71	F	2	400	None	0	29	18	25

^a^Signifies a change in symmetry between OFF and ON; bolding of subject number represents a failure to achieve a minimal clinical difference 1 hour after DRT administration

### Differentiation of PD subjects OFF medication from healthy controls

All subjects were able to complete the *object hit task* and the *object hit and avoid task* in both “OFF” and “ON” drug states. Table 2 compares their robotic task performance OFF DRT to the performance of healthy controls. In the *object hit task*, mean hand speed was 41% slower, movement area was 31% smaller, and subjects hit 25% fewer targets than controls. In the *object hit and avoid task*, mean hand speed was 47% slower, movement area was 40% smaller, and 23% fewer targets were hit. ROC analysis for each parameter is listed in Table 2.

**Table 2:** Comparison of PD subjects KINARM task OFF DRT performance to (1) healthy controls and (2) UPDRS ratings

PD sign	KINARM Task	Task parameter	Subject performance compared to controls (statistical test)	ROC AUC (95% confidence interval)	Performance correlated^a^ to UPDRS subscore^b^
Bradykinesia	Object hit task	Mean hand speed	41% slower (MW, U = 365, *P* < 0.001)	0.94 (0.90–0.98)	c.c. = − 0.615, *P* < 0.001
		Movement area	31% smaller (Student t-test, t = 6.7, *P* < 0.001)	0.84 (0.76–0.92)	c.c. = − 0.411, *P* < 0.05
		Total hits	25% less targets (MW, U = 642, *P* < 0.001)	0.90 (0.85–0.95)	n.s. (*P* = 0.27)
	Object hit and avoid task	Mean hand speed	47% slower (Student t-test, t = 9.6, < 0.001)	0.93 (0.87–0.98)	c.c. = − 0.633, *P* < 0.001
		Movement area	40% smaller (Student t-test, t = 8.9, < 0.001)	0.91 (0.85–0.97)	c.c. = − 0.594, *P* < 0.01
		Total hits	23% less targets (Student t-test, t = 8.7, < 0.001)	0.88 (0.81–0.96)	n.s. (*P* = 0.055)
		Distractor hits	n.s. (MW, U = 1962, *P* = 0.276)	n.s. (0.45–0.69)	n.s. (*P* = 0.102)
Rigidity	Passive stretch task	Start total torques	n.s. (Student t-test, t = − 1.696, *P* = 0.096)	n.s. (0.48–0.80)	n.s. *P* = 0.09)
		End total torques	34% lower (Student t-test, t = − 6.245, < 0.001)	0.91 (0.82–0.99)	c.c. = − 0.464, *P* < 0.05
		Total torque difference	64% higher (Student t-test, t = −4.844, *P* < 0.001)	0.81 (0.69–0.94)	n.s. (*P* = 0.45)
		Hold torque	3.6 times greater (MW, U = 219, *P* < 0.05)	0.68 (0.52–0.83)	n.s. (*P* = 0.20)
		Peak speeds	9% slower (MW, U = 118, *P* < 0.001)	0.83 (0.71–0.94)	n.s. (*P* = 0.25)
Postural stability	Unloading task	Deceleration time (ms)	9% faster (Student t-test, t = − 2.305, *P* < 0.05)	0.66 (0.53–.80)	n.s. (*P* = 0.42)
		Maximum displacement	29% shorter (MW, U = 277, *P* < 0.01)	0.71 (0.57–0.85)	n.s. (*P* = 0.69)
		Return time	n.s. (MW, U = 452, *P* = 0.75)	n.s. (0.37–0.68)	n.s. (*P* = 0.35)
		Endpoint error	n.s. (MW, U = 447, *P* = 0.70)	n.s. (0.38–0.68)	n.s. (*P* = 0.23)

^a^Spearman Rank Order Correlation, *n.s.* not significant, *MW* Mann-Whitney Rank Sum Test, *c.c.* Spearman Rank Order Correlation, ^b^UPDRS subscore for bradykinesia score used items 23–26 (maximum score = 32), UPDRS subscore for rigidity score used item 22 (maximum score = 20), and UPDRS subscore for posture stability score used item 30 (maximum score = 4)

**Fig. 2:**
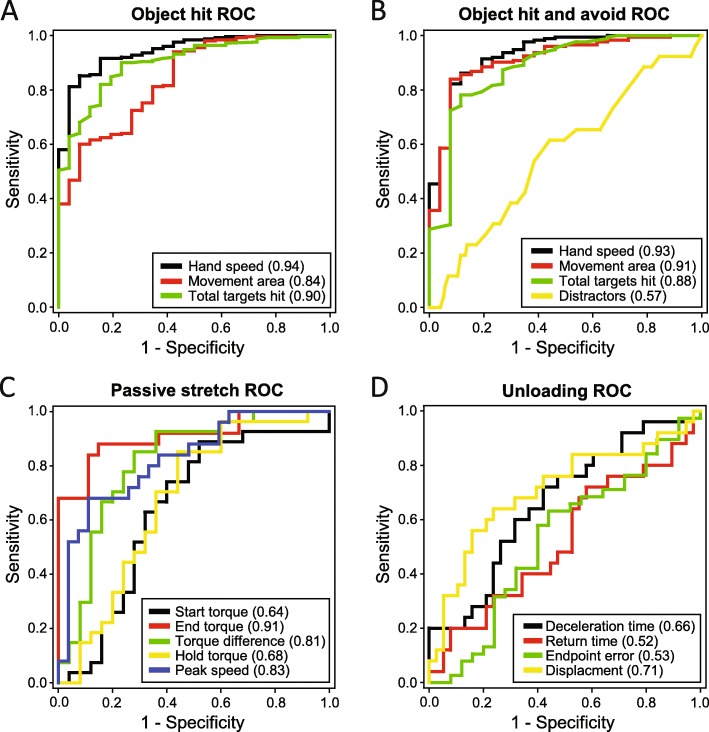
Differentiating Controls and PD subjects using ROC Analysis*.* ROC analyses conducted on robotic parameters for the **a** *object hit task*, **b** *object hit and avoid task*, **c** *passive stretch task* and **d** *unloading task* between controls and subjects with PD. The AUC for each parameter is denoted in the brackets). ROC = Receiver operating characteristic. AUC = area under the curve

ROC analysis comparing how robotic task performance parameters discriminated subjects OFF DRT from controls for the *object hit task* and *object hit and avoid task* are displayed in Fig. 2a and b, respectively. The parameter with the highest predictive accuracy was the mean hand speed in the *object hit task* with AUC = 0.94 and in the *object hit and avoid task* with AUC = 0.93. There was no statistical difference between the AUCs for mean hand speed between these tasks (Chi-square test, χ^2^ (1, *N* = 200) = 0.17, *P* = 0.68) indicating that the addition of distractors did not improve the predictive accuracy of mean hand speed in differentiating subjects with PD from controls.

One of the 26 subjects with PD was not able to perform the *passive stretch task* with one arm during the OFF condition due to discomfort (but was able to perform with both hands in the ON condition) and was excluded from all of the rigidity analyses. This subject’s UPDRS rigidity score ratings in the OFF DRT state were right arm = 0, right leg = 1, left arm = 1, left leg = 1, and neck = 0 (total OFF UPDRS rigidity score = 3). In comparison to the total OFF UPDRS rigidity score of the group, three other subjects scored 3, one scored a 2, and the rest were 4 or greater (median = 7). End total torques were 34% lower, the total torque difference (defined as difference between total start and total end torque per subject) was 64% higher, hold torque was 3.6 times greater, and peak speeds were 9% slower in the subjects with PD versus controls (Table 2). Figure 2c shows the ROC for these parameters where the highest predictive accuracy was end total torque with AUC = 0.91 (95% Confidence Interval: 0.82–0.99).

The same subject that could not complete the *passive stretch task* did not perform the *unloading task.* Deceleration time (ms) after force unloading was 9% faster and maximum displacement was 29% shorter in subjects with PD than controls (Table 2). Figure 2d shows ROC for these parameters and return time and endpoint error. The highest predictive accuracy of a parameter was maximum displacement with AUC = 0.71 (95% Confidence Interval: 0.57–0.85).

### Task performance correlations to OFF UPDRS

The subjects mean UPDRS Part III scores OFF medication was 29.7 (SD = 10.6). Their UPDRS ratings correlations to robotic task performance is shown in Table 2 (right most column). The mean UPDRS bradykinesia score OFF DRT was 12.6 (SD = 5.4) (items 23–26, maximum score = 32). The best correlation with UPDRS bradykinesia scores was for mean hand speed in the *object hit task* and *object hit and avoid task.* Correlations of just upper extremity UPDRS bradykinesia scores (items 23–25, maximum score = 24) with hand speeds were not as highly significant (Spearman Rank Order Correlation, Correlation Coefficient = − 0.5 and − 0.588, *P* <  0.01 for each task). The mean rigidity UPDRS score OFF DRT was 6.7 (SD = 3.2) (item 22; maximum score = 20). UPDRS rigidity scores were best correlated with end total torques in the *passive stretch task*. There was no significant correlations with only upper extremity rigidity (maximum score = 8) (Spearman Rank Order Correlation, *P* = 0.21). None of the 4 parameters in the *unloading task* were correlated to the UPDRS posture stability score (item 30, maximum score = 4).

### Asymmetry bimanual tasks

**Fig. 3:**
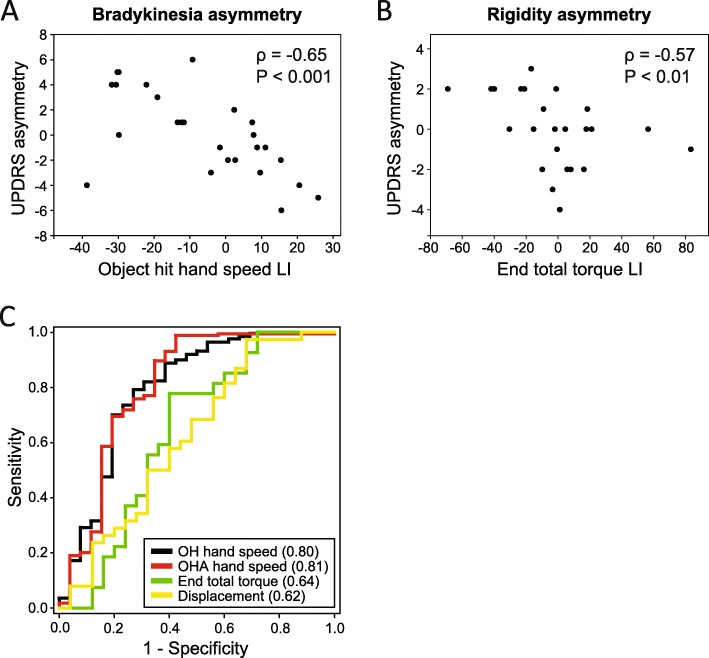
Comparison of side to side asymmetry between KINARM parameters and UPDRS scores. Correlations were conducted between robotic laterality indices and UPDRS right minus left side score differences for **a** bradykinesia asymmetry and **b** rigidity asymmetry. **c** ROC analysis of laterality indices in different tasks between PD subjects and controls. LI = laterality index

Correlations to UPDRS score asymmetries were performed with single robotic parameters that displayed the highest ROC areas. Laterality indices for hand speed in *object hit task* and *object hit and avoid task*, and end total torques in the *passive stretch task* were compared to UPDRS score asymmetries in subjects in the OFF condition. Figure 3a shows a correlation between the laterality index for *object hit task* hand speed and UPDRS bradykinesia score asymmetries (Spearman Rank Order Correlation, correlation co-efficient = − 0.65, *P* < 0.001). Similarly, *object hit and avoid task* hand speed laterality index correlated with the UPDRS bradykinesia score asymmetries (Spearman Rank Order Correlation, correlation co-efficient = − 0.666, *P* < 0.001) (not shown). There was a significant correlation between the most affected side for rigidity and laterality index for end total torque (Spearman Rank Order Correlation, correlation co-efficient = − 0.566, *P* < 0.01) (Fig. 3b). ROC analysis revealed that the only parameter that discriminated subjects from controls was hand speed asymmetry in either the *object hit task* and *object hit and avoid tasks* (significant ROC curve areas = 0.80 and 0.81, respectively; 95% confidence interval 0.68–0.92, *P* < 0.0001) (Fig. 3c).

### Drug effects

**Fig. 4:**
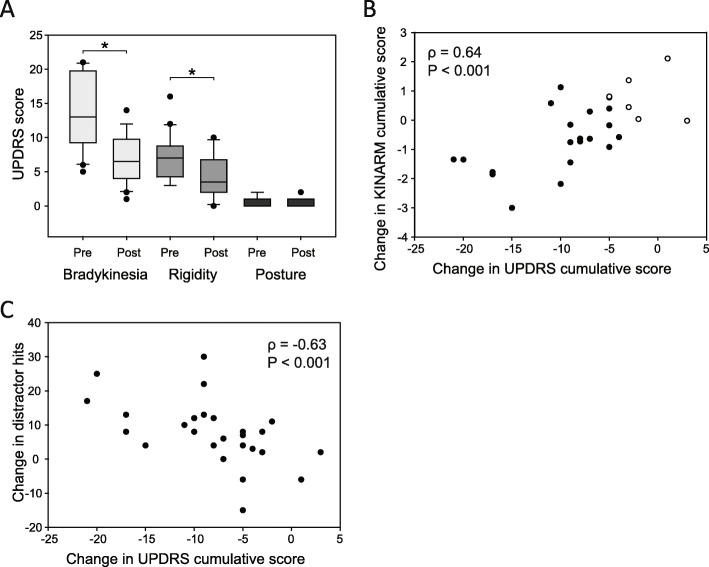
DRT effects on UPDRS scores and KINARM performance. **a** The change in UPDRS scores for bradykinesia, rigidity, and posture with DRT in 20 subjects that displayed a minimal clinical difference (* *p* < 0.001). **b** The correlation between the change in KINARM cumulative score (calculated from Z-scores for selected parameters) and the change in UPDRS cumulative score for bradykinesia, rigidity, and posture due to the administration of DRT for all subjects. The open white circles represent the 6 subjects that didn’t meet the criteria for minimal clinical difference. **c** Correlation between the change in the number of distractor hits and the change in UPDRS cumulative scores due to the administration of DRT

A minimal clinical improvement [16] was defined as a reduction in the total UPDRS part 3 of greater than six points. This minimal clinical difference was observed in 20 of 26 subjects after DRT. This cut off was used to ensure a clinical effect of DRT because the dose of levodopa was not uniform across all subjects and each likely had a different sensitivity to the drug. The total UPDRS part 3 score in these 20 patients decreased from 32 to 16 (Wilcoxon Signed Rank Test, Z = − 3.933, *P* < 0.001). Bradykinesia UPDRS score decreased from 13 to 6.5 (Wilcoxon Signed Rank Test, Z = − 3.925, *P* < 0.001), rigidity decreased from 7.1 to 4 (paired t-test, t = 8.498, *P* < 0.001), but there was no change in postural stability (median = 0) (Wilcoxon Signed Rank Test, Z = − 2.121, *P* = 0.063)(Fig. 4a).

The summary data for the effects of DRT on robotic parameters in the 20 subjects with the minimal clinical improvement is shown in Table 3. In *object hit and avoid task*, (but not the *object hit task*), mean hand speed increased by 28% with DRT. Total hits increased due to DRT administration in both *object hit task* (median 163 to 179) and the *object hit and avoid task* (mean 97 to 106). End total torque in the *passive stretch task* was not changed by medication. Maximum displacement was 11% greater in the *unloading task* (and closer to healthy control performance) following medication.

**Table 3:** (1) The effect of DRT on KINARM parameters and (2) correlating the effects of DRT between UPDRS scores and KINARM tasks

UPDRS	KINARM Task	Parameter	Effect of DRT on KINARM parameter (*n* = 20)	correlation with DRT changes in UPDRS; co-efficient (P) (*n* = 26)^a^
Bradykinesia	Object hit task	Mean hand speed	n.s. (paired t-test, t = − 1.667, *P* = 0.112)	− 0.443 (*P* < 0.05)
		Total hits	median 163 to 179 (Wilcoxon Signed Rank Test, Z = 2.278, *P* < 0.05)	−0.162 (*P* = 0.42)
	Object hit and avoid task	Mean hand speed	increased by 28% (paired t-test, t = − 2.130, *P* < 0.05)	−0.609 (*P* < 0.001)
		Total hits	mean 97 to 106 (paired t-test, t = − 2.806, *P* < 0.05)	−0.519 (*P* < 0.01)
		Distractor hits	17 to 26 (paired t-test, t = − 3.869, *P* = < 0.001)	−0.629 (*P* < 0.001)
Rigidity	Passive stretch task	End total torques	n.s. (Wilcoxon Signed Rank Test, Z = 0.241, *P* = 0.83)	0.043 (*P* = 0.84)
Postural stability	Unloading task	Maximum displacement	11% greater (paired t-test, t = − 2.377, *P* < 0.05)	−0.17 (*P* = 0.41)

^a^Spearman Rank Order Correlation, *n.s.* not significant

All 26 subjects were used to correlate the change in UPDRS scores and robotic parameters after DRT in order to determine if both tests were consistent in showing larger improvements for the minimal clinical difference groups and no improves/small improvement for those who did not display a minimal clinical difference. The total UPDRS part 3 score in all 26 patients decreased from 30 to 16 (paired t-test, t = 7.812, *P* < 0.001). In *object hit task*, the increase in mean hand speed was correlated to the decrease in UPDRS bradykinesia scores (Table 3). In *object hit and avoid task*, the correlations were more significant than the *object hit task* with the increase in mean hand and total hits being highly correlated to the decrease in UPDRS bradykinesia scores. For the 25 subjects with PD that were able to complete the *passive stretch task*, rigidity changes quantified by end total torques were not correlated to UPDRS improvement. Similarly, the change in maximum displacement in the *unloading task* was not correlated to UPDRS improvement.

### Cumulative score

In the 20 subjects that exhibited minimal clinical differences in total UPDRS part III scores, the UPDRS cumulative score (sum of bradykinesia, rigidity, and posture items) decreased from 21 to 11 (paired t-test, t = 7.833, *P* < 0.001). The robotic cumulative score (the root-sum-square distance of the Z-scores) was used as a measure to directly compare the combined performance on all 4 tasks with UPDRS score. The robotic parameters Z-scores that exhibited a significant change (*P* < 0.05) following DRT were number of target hits in *object hit task*, the number of target hits, mean hand speed, movement area in *object hit and avoid task*, hold torque in the *passive stretch task*, and maximum displacement in the *unloading task*. In these 20 subjects the robotic cumulative score was 4.5 OFF medication and decreased to 3.8 ON medication (paired t-test, t = 3.041, *P* < 0.01). There was a significant correlation between robotic cumulative score and the sum of UPDRS scores for bradykinesia, rigidity, and posture stability for all 26 subjects (Spearman Rank Order Correlation, Correlation Coefficient = 0.64, *P* < 0.001) (Fig. 4b). The six subjects that did not meet the criteria for minimal clinical difference using the total UPDRS part 3 can be observed in the top right of the figure (open white circles).

### Task errors

The number of distractor hits in the *object hit and avoid task* was not different between OFF medication subjects and healthy controls (Table 2). The number of distractors hit in the *object hit and avoid task* was increased with DRT from 17 to 26 in the 20 subjects with the minimal clinical improvement (Table 3) and from 18 to 26 in all 26 subjects (paired t-test, t = − 4.150, *P* = < 0.001). This increase was elevated compared with healthy controls (Mann-Whitney Rank Sum Test, U = 1121, *P* < 0.001). In all subjects, the DRT related increase in distractor hits was significantly correlated to the reduction in the sum of UPDRS bradykinesia, rigidity, and posture scores (Spearman Rank Order Correlation, correlation co-efficient = − 0.63, *P* < 0.001) (Fig. 4c). No correlations were observed with MoCA scores (Spearman Rank Order Correlation, correlation co-efficient = − 0.250, *P* = 0.215), levodopa dose (Spearman Rank Order Correlation, correlation co-efficient = 0.110, *P* = 0.590), or years with PD (Spearman Rank Order Correlation, correlation co-efficient = 0.248, *P* = 0.219)].

## Discussion

The current study demonstrates the utility of integrated robotic assessment of the multiple signs of PD. The platform was able to differentiate subjects with PD from healthy controls based on upper limb bradykinesia, rigidity, postural stability, and limb asymmetry metrics. It was able to be used by subjects with both strong and weak motor symptoms (as per their high and low UPDRS scores) and no floor or ceiling effects were encountered. The ROC analysis demonstrated high distinguishability between PD subjects and controls in all four robotic tasks and was able to separate PD subjects from controls using a simple laterality index. After the administration of DRT, both individual robotic parameters and the robotic cumulative scores moved closer to healthy control values. There was a significant correlation between the robotic cumulative scores and UPDRS cumulative scores after DRT administration.

Our study supports the use of technologies that quantify multiple parkinsonian signs into a summary score to accurately differentiate subjects from healthy controls [1]. In the UPDRS assessments in the present study, cumulative scores were more sensitive to the effects of DRTs. In the case of bradykinesia and mean hand speed, the most significant correlations were observed when the full bradykinesia score was compared (items 23–26) rather than just upper extremity scores. UPDRS rigidity and end total torque were correlated when the full rigidity score was used (item 22; maximum score = 20) rather than just the upper limbs indicating that adding multiple items in the UPDRS increases the power of this clinical scale in quantifying the signs of PD. Similarly, we found that the robotic cumulative score was more sensitive to the effect of DRT rather than individual Z-score parameters. The highest predictive accuracy was given by mean hand speed (AUC = 0.94 in object hit task) indicating that no single task completely separated all subjects with PD from healthy control performance. There were likely some healthy controls who performed poorly on individual tasks and were indistinguishable from subjects with mild disease that performed well (for patients with PD). This is consistent with clinical reasoning. A patient may move slightly slower than normal for a variety of reasons such as neurological, musculoskeletal, age, medications, task complexity etc. But if any patient also has mild rigidity, rest tremor, and asymmetry these together may diagnose PD. This suggests that the robotic cumulative score, similar to the UPDRS Part 3, rather than individual tasks is better to objectively and accurately track the progression and treatment of clinically relevant parkinsonian signs [13].

### Bradykinesia

In both the *object hit task* and *object hit and avoid task*, mean hand speed and limb asymmetries showed high discriminatory abilities in differentiating subjects with PD from healthly controls. Our findings support similar observations of mean hand speed by Wiratman et al. [43] using the KINARM *object hit and avoid task*. Mean hand speed and limb asymmetries had significant correlations with UPDRS bradykinesia scores when subjects were OFF medication. Although we expected the introduction of a higher cognitive load using distractors in the *object hit and avoid task* would substantially increase the task’s discriminatory ability [35], there was no significant effect. However, the effects of DRT on bradykinesia were more significant in the *object hit and avoid task* than in the *object hit task.* The administration of DRTs has been implicated in increased cognitive flexibility in PD [9] and DRTs have been shown to improve Parkinsonian performance on tasks with higher cognitive loads, when compared to their OFF performance. For example, Costa et al. [9] reported significant improvements in Parkinsonian performance on tasks that required higher flexibility measures (i.e. interference condition of the Stroop task) after DRT administration.

### Rigidity

Similar to bradykinesia, the robotic platform was able to differentiate rigidity in subjects with PD compared to healthy controls. The parameter “end total torque” in the *passive stretch task* displayed a higher discriminatory ability in the differentiation of PD from healthy controls. Furthermore, the quantification of PD rigidity using end torque values was correlated to the UPDRS OFF medication scores. The single subject who was unable to complete rigidity testing had a low clinical rigidity score. This suggests that the severity of clinical rigidity does not limit testing with the exoskeleton. The most affected side that was identified by the UPDRS OFF medication (right minus left side score difference within UPDRS item 22) was correlated with greater end total torque in the *passive stretch task*. However, contrary to the change in UPDRS rigidity scores with DRT, none of the parameters from the *passive stretch task* showed a change following DRT administration. These discrepancies are likely due to several factors. First, both neurologists were not blinded to the subject’s medication state and could have overestimated the change in rigidity. Since clinicians are aware of the subject’s medication state and familiar with what is expected to be seen in that given state, they can be prone to rely on their previous experiences and introduce a level of bias [44]. Second, end total torque may not be the optimum way to quantify rigidity and could be improved by development of a rigidity task score that encompasses many parameters during this task. Third, the two methodologies for rigidity testing also differ in that the clinicians tested multiple joints in the upper limb whereas the robot only tested the elbow.

By constraining the subjects upper extremities, the robot can move the arm to reach a peak velocity and produces a consistent measure of the torque needed to move and stop the limb. Our study supports measuring rigidity with the use of servo-motors [31] and similar technologies, like a supported test rig [39] or haptic technology [5], to passively move a joint through flexion and extension motions. Parameters such as torque [39], resistive torque [31] and angular impulse [14] are used to quantify increases in muscle tone and the position of the elbow joint during a flexion-extension movement and are highly correlated with the UPDRS. While handheld force sensors that are coupled with the rater’s passive movement of a limb at the joint can be used [22, 32] there is less control of limb velocity which can cause discrepancies in the measured tone. Electromyography recordings can be implemented to avoid constraining patients and have shown to correlated with enhanced stretch-related increases in activity [26]. However, as mentioned by Park et al. [29] the accuracy of EMG recordings depends on the condition of electrodes and soft tissue, which is responsible for the production of weak correlations between UPDRS and EMG-rigidity measures.

### Posture

The *unloading task* was able to differentiate PD subjects from healthy controls where maximum displacement was 29% shorter in subjects OFF medication in comparison to controls. This may be due to increases in rigidity after the > 12 h DRT washout period where the constant random force released may not have ben large enough to overcome the increased antagonist muscle tone. One hour after DRT administration, subjects limbs were not as rigid therefore producing maximum displacement values that were greater than OFF values and were more similar to healthy controls. This is in contrast to the UPRDS scale which did not show a DRT effect on postural stability. This discrepancy may be due to the UPDRS scale’s predictable quantification of postural instability. Item 30 of the UPDRS measures postural instability using the pull test. Patients are ready to receive a destabilizing pull from clinicians in which they are expected to recover. Prior to the pull patients stiffen their bodies to meet the force of the clinician, which can confound the postural results. Furthermore, the item does not consider how far a patient is displaced or how quickly they react to the pull. Factors such as postural sway, which assesses changes in an individual’s centre of gravity can also be used to quantify a patient’s tendency to fall or balance [2]. In particular, abnormalities in postural sway can also be a reliable measure for assessing changes in postural instability in the PD population [2] and can be measured by a portable device like a tablet or smartphone [12, 28]. The KINARM Endpoint variant with an adjustable height does have the ability to measure postural sway by having the subject perform the task in a standing position with the addition of force plates on the robotic platform [11, 23]. Importantly, a comparison between upper limb postural stability as measured with the KINARM to other methods of gait and posture analysis should be performed.

### Task errors

The increase in distractor hits in the *object hit and avoid task* reflects a measure of worsening sensorimotor control after DRT administration when compared to healthy controls or the OFF medication state. This increase was inversely correlated with a reduction in UPDRS cumulative scores. The increase in distractor hits relative to controls is consistent with a recent study by Wiratman et al. [43] using the KINARM that demonstrated that patients with poor performance on standard cognitive batteries had higher distractor hits. In the present study no correlations were observed with MoCA scores but this may be due to limitations in using the MoCA to quantify cognitive function in subjects with PD. These observations highlight that robotic technology is useful to objectively quantify cognitive dysfunction in addition to motor performance.

In contrast to Wiratman et al. [43] we found an effect of DRT on distractor hits whereby patients that had a larger reduction on the cumulative UPDRS score with DRT hit more distractors (see Fig. 4c). This increase in task errors may be due to effects of DRT on impulsivity [33]. Dopamine’s role in impulsivity has been correlated with the D2/D3 autoreceptor in the substantia nigra/ventral tegmental area [6]. A lower D2/D3 receptor availability is also associated with enhanced dopamine release in the striatum [6]. In PD, DRTs exert their effects by directly activating D1 and D2 receptors over a persistent but non-physiological period of dopamine release [19]. Although beneficial at alleviating motor symptoms of PD, the persistent stimulation has been hypothesized to produce an “overdose” of ventral striatal-cortical stimulation, where consequences manifest as impulsive control disorders [42].

### Limitations

The primary limitation of our study is the non-blinding and randomization of medicated vs. non-medicated states for UPDRS assessment. Both clinicians were aware that the subject was OFF or ON medication and this may have introduced clinical bias [20]. Lastly, we were not able to quantify tremor or dyskinesia due to the physical constraints of the exoskeleton arms. Rest tremor is a primary sign of PD and dyskinesia is a potentially debilitating side effect of medication [21]. Inclusion of these signs into the robotic battery would improve its sensitivity and specificity for PD that may be possible with the KINARM Endpoint robot or related technologies that have force sensors in the handle grasped by the subject. In addition, the combination of parameters that were used to calculate the robotic cumulative score could not be directly compared to the controls due to the fact that different control subjects performed different sets of tasks. Given that there were greater differences between healthy controls and subjects with PD it is likely that a robotic cumulative score for healthy subjects perform all the tasks would better differentiate subjects with PD from healthy controls.

## Conclusion

The current study validates the use of the integrated robotic technology to quantify the signs of bradykinesia, rigidity and limb postural instability of PD. Similar to the addition of items in the UPDRS, we found that the robotic cumulative score was more sensitive to the effect of DRT in comparison to individual robotic parameters. This platform has the potential to be used a diagnostic tool to differentiate subjects with PD from healthy individuals and to objectively quantify the effect of dopaminergic medication and other therapies.

## Supplementary information

**Additional file 1.** Comparison of rigidity parameters (start/end total torques, hold torque, and peak speeds) derived from the *passive stretch task* performed over 600 ms and 1500 ms. A greater number of rigidity parameters were abnormal (i.e. failed in comparison to healthy subject performance) when faster stretches (600 ms) were used.

## Data Availability

The datasets used and analysed during the current study are available from the corresponding author upon reasonable request.

## Abbreviations

DRT: Dopamine replacement therapies
MoCA: Montreal Cognitive Assessment
PD: Parkinson’s Disease
ROC: Receiver operating characteristic
UPDRS: Unified Parkinson’s Disease Rating Scale
