# Supplementary figures and images for: Integrated robotics platform with haptic control differentiates subjects with Parkinson’s disease from controls and quantifies the motor effects of levodopa
Source: J Neuroeng Rehabil. 2019 Oct 26;16:124. doi: 10.1186/s12984-019-0598-5 (PMC6815040; doi:10.1186/s12984-019-0598-5)

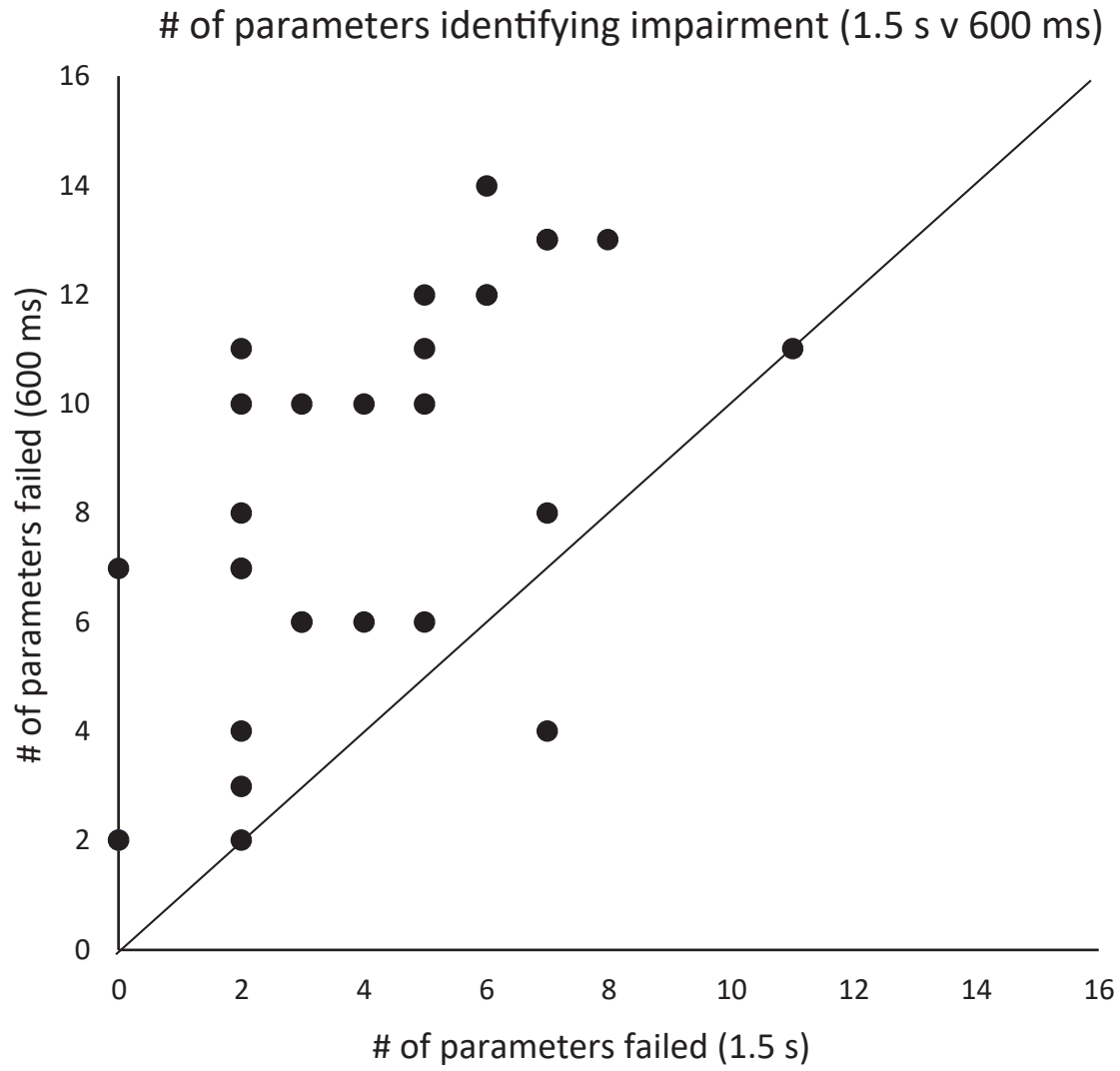

Supplement: Supplementary file 1 — Additional file 1. Comparison of rigidity parameters (start/end total torques, hold torque, and peak speeds) derived from the passive stretch task performed over 600 ms and 1500 ms. A greater number of rigidity parameters were abnormal (i.e. failed in comparison to healthy subject performance) when faster stretches (600 ms) were used. [file 12984_2019_598_MOESM1_ESM.pdf]
